# Nanotechnology Revolutionizing Food Processing Technology

**DOI:** 10.3390/foods15040643

**Published:** 2026-02-10

**Authors:** Zhifei Gou, Weiyun Guo, Ting Du, Sijie Liu, Yuechun Li, Jianlong Wang, Wentao Zhang, Jihong Huang

**Affiliations:** 1 School of Food and Pharmacy, Xuchang University, Xuchang 461000, China; gzf@nwafu.edu.cn (Z.G.); gwy2002@126.com (W.G.); 2 College of Food Science and Engineering, Northwest A&F University, Xianyang 712100, China; duting970520@nwafu.edu.cn (T.D.); sijieliu@nwafu.edu.cn (S.L.); yuechunli@nwsuaf.edu.cn (Y.L.); wanglong79@nwsuaf.edu.cn (J.W.); 3 Collaborative Innovation Center of Functional Food by Green Manufacturing, Xuchang 461000, China; 4 College of Agriculture, Henan University, Kaifeng 475004, China

**Keywords:** nanotechnology, food supply chain, food processing, food processing technology, sustainable food processing, synergistic effect, additive effect

## Abstract

Owing to population expansion, widespread diseases and pandemics, climate alterations, and evolving consumer preferences, the optimization of production processes and technological advancements in food processing have become imperative. The integration of nanotechnology with food processing technology, characterized by numerous advantages, holds the promise to establish a secure, efficient, and sustainable food supply system. Nanoparticles can mitigate the risk of microbial contamination through the generation of reactive oxygen species and by leveraging their electrical charge properties to exert antibacterial effects or detoxify; they can serve as an energy transfer medium to enhance food quality; or utilize its high catalytic efficiency for the recycling and decomposition of food waste. When integrated with food processing technologies, they demonstrate a synergistic or additive effect. This paper reviews representative instances of the convergence between nanotechnology and food processing technologies, elucidates the practical application effects and underlying mechanisms, aims to inform the development of more advantageous application strategies for nanotechnology in the realm of food processing.

## 1. Introduction

The global food system is confronting profound challenges influenced by a multitude of factors, including world population growth, wars and frequent geopolitical conflicts, the impact of diseases (such as COVID-19), and climate variability [1]. These factors collectively exacerbate food shortages and security issues. Over 820 million individuals suffer from poverty, hunger, and malnutrition, unless timely and effective response programs are devised and executed, the situation is projected to worsen as the global population is anticipated to reach 10 billion by 2050 [2]. The various adverse outcomes underscore the fragility of the existing food supply chain, thereby necessitating the development of a green, resilient, and sustainable food supply chain for the future [2].

As the core of the supply chain, food processing generates trillions of dollars in annual revenue globally and represents a critical target for supply chain optimization. To address these challenges and align with evolving consumer preferences, various food processing technologies offer new opportunities for building high-quality processing systems, such as microwave (MW), pulsed electric fields (PEF), ultrasound and cold plasma (CP) technologies. They are characterized by efficient, low-waste processing and the provision of safe, nutritious finished products. For instance, PEF has been utilized to extract bioactive compounds from food waste, thereby reducing resource waste [3]. Additionally, CP has shown potential in safe and sustainable food production through applications like pesticide degradation, enhanced seed germination rates, and food antimicrobial procedures [4]. However, the application of these technologies is not without limitations, as some can have adverse effects on food, such as local overheating caused by MW, ultrasound-induced oxidation of food [5].

To mitigate these drawbacks and enhance the favorable application of food processing technologies, we propose the integration of nanotechnology. Since the inception of the United States National Nanotechnology Initiative (NNI) in 2000, nanomaterials have rapidly advanced in various fields (agriculture, medicine, food, and biology) due to their high specific surface area, high reactivity, and customizable design [6]. In the field of food science, nanotechnology has demonstrated significant potential, particularly in areas such as active packaging, nutritional preservation, and food quality sensors. These applications typically exploit the inherent properties of nanomaterials, such as their high surface activity and versatile design possibilities. This includes, but is not limited to, packaging matrices where nanoscale particles serve as the primary active components, coatings applied to food surfaces for antimicrobial, antioxidant, and UV-blocking purposes [7,8,9,10]; and the use of nanomaterials in colorimetric, immunological, and electrochemical sensors to monitor changes in food quality, thereby ensuring food safety [11,12,13]. A growing body of research is developing diverse nanomaterial combinations and exploring broader application scenarios, including integration with food processing technologies. Unlike the effects achieved by either approach alone, the combined use of nanotechnology and food processing techniques often results in synergistic or additive effects that are unattainable by either component independently.

The integration of nanotechnology with food processing technology offers several advantages: (i) The inherent high efficiency of nanomaterials, combined with their synergistic or additive effects when integrated with processing technologies, can effectively optimize manufacturing processes and improve product quality. (ii) The incorporation of nanotechnology can mitigate the potential adverse effects of processing technologies on food products, such as reducing ultrasonic intensity to prevent lipid oxidation. (iii) Avoidance of contamination risks associated with complex process operations, compared to other improvement methods (e.g., adding hot air, far-infrared, and stirring processes in MW heating). These multiple positive effects demonstrate the potential of this integration in food processing.

This paper presents the applications and fundamental mechanisms of nanotechnology-combined food processing techniques in the food industry, with classification of their application ranges (Table 1). Our objective is to facilitate more advantageous applications of nanotechnology in food processing through modular process optimization, thereby supporting the development of safe, efficient, and sustainable food supply systems.

## 2. Positioning and Methods of This Review

The advancements in nanotechnology applications within the food sector have been extensively documented. The resulting focused research hotspots can be broadly divided into two categories: first, those based on differences in nanoparticles or their matrices, such as micro/nanomotors, nanozymes, and essential oil/emulsion systems based on nanomaterials [85,86,87,88,89,90]; second, those based on variations in application areas, for example, food quality monitoring, food safety control, and degradation of hazardous substances [91,92,93,94]. Additionally, substantial literature elaborates on stage-specific innovative progress related to particular food processing technologies [95,96,97,98,99]. However, a knowledge gap exists in the systematic summarization of studies that integrate specific nanomaterials with food processing techniques. This review aims to provide a unique panoramic overview by examining representative studies centered on core technologies, such as radio frequency, MW, and ultraviolet (UV) irradiation. By doing so, it seeks to consolidate scattered research findings, clarify the principles underlying the synergy between nanomaterials and processing technologies, and ultimately guide the design of more efficient and sustainable food processing methods.

This narrative review was conducted following a structured approach to comprehensively explore the synergistic and additive effects between nanotechnology and various food processing technologies. The literature search was designed to identify high-quality scientific evidence relevant to applications in food preservation, safety, quality control, and green processing.

Relevant literature was systematically retrieved from two major scientific databases: Web of Science (Core Collection) and PubMed. The final search was completed in September 2025. A comprehensive search strategy was employed using a combination of free-text keywords and Boolean operators (AND, OR). The key search terms encompassed: (1) specific food processing technologies (e.g., “radio frequency”, “microwave”, “ultraviolet”, “cold plasma”, “ultrasound”); (2) core nanotechnology terms (e.g., “nanotechnology”, “nanomaterial”, “nanoparticle”); and (3) target application outcomes (e.g., “food preservation”, “food safety”, “food quality”, “green processing”, “sterilization”). Representative search strings included: (“nanoparticle” OR “nanomaterial”) AND (“microwave” OR “radio frequency”) AND (“food preservation” OR “food safety”). We distinguish between food preservation and food safety, as they are primarily attributed to spoilage microorganisms and pathogenic bacteria, respectively. Consequently, during the literature screening process, we categorized studies based on the authors’ descriptions of the mechanisms and effects of synergistic or additive interactions. Furthermore, in the subsection on UV irradiation technology, we excluded literature that focused on employing nanotechnology to shield the food matrix from adverse reactions induced by UV exposure.

The review primarily focused on peer-reviewed research articles and highly relevant reviews published in English. To capture the most recent advancements, priority was given to publications from the last 6 years (2020–2025). However, earlier representative and seminal studies were also included to provide necessary context and a more complete discussion. Studies were selected if they investigated the integration of nanomaterials with food processing technologies for applications directly related to the food supply chain. While research employing real food matrices was prioritized, studies on relevant model systems or mechanisms with clear implications for food applications were also considered. Publications exclusively focused on non-food applications (e.g., environmental remediation, pharmaceuticals) without a direct link to food processing were excluded.

Retrieved records were initially screened based on titles and abstracts to assess relevance. Full texts of potentially eligible articles were then reviewed. Key information from the selected literature, including the processing technology, nanomaterial type, food sample, application method, main findings, and reference, was extracted, synthesized, and is summarized in Table 1. The narrative synthesis was organized by food processing technology to provide a clear, technology-centric analysis of the convergent effects with nanotechnology.

## 3. Radio Frequency Combined with Nanotechnology

Radio frequency (RF), an electromagnetic wave ranging from 300 kHz to 300 MHz. It offers several advantages, including rapid heating, low cost, and significant penetration depth. The primary mechanism behind its thermal effects is attributed to the rapid molecular movement induced by high-frequency electromagnetic waves. This technology has found commercial application in various stages of food processing, such as thawing, drying, deworming, and sterilization [100].

ZnO nanoparticles exhibit remarkable antibacterial properties by disrupting microbial structures through the release of Zn^2+^ ions and reactive oxygen species (ROS). Furthermore, the level of antibacterial activity is typically inversely correlated with particles size [101,102]. Xu et al. immersed carrots in a suspension of ZnO nanoparticles with diameters ranging from 1 to 100 nm and combined with RF processing, resulting in an extended shelf life of up to 60 days. The total colony-forming unit (CFU) remained below 3 log CFU/g, demonstrating superior efficacy compared to either treatment alone [14]. The research team further explored the application of ZnO nanoparticles for preserving various Chinese dishes, all exhibiting effective bactericidal action alongside lower product quality degradation than high pressure steam sterilization (HPS) methods [103,104]. This synergistic effect may result from radiofrequency-induced sublethal bacterial damage, which enhances the efficacy of antimicrobial agents [105]. In this instance, Zn^2+^ and ROS produced by ZnO may more readily affect microorganisms via cell membranes.

The integration of carbon dots (CDs) and RF technology is regarded as an effective antibacterial approach. Zhao et al. integrated CDs with RF to pasteurize boiled gansi dishes (a traditional Chinese dish), demonstrating they could significantly inactivate *Escherichia coli* (*E. coli*), *Staphylococcus aureus* (*S. aureus*), and *Bacillus subtilis*. The treatment lasted 16 min, the count of *Bacillus subtilis* decreased by 4.63 log CFU/g; and compared with HPS, the adverse effects on product quality characteristics are reduced [15]. Zhao et al. incorporated CDs into polyvinyl alcohol (PVA) films, which enhanced the mechanical tensile properties and thermal stability of the films. When combined with RF, they demonstrated synergistic Oregano antibacterial effects. In their study, fried meatballs were first vacuum sealed with the CDs film packaging, then subjected to 20 min of radiofrequency treatment with 20 mm plate spacing. This combined approach effectively extended the shelf life of the meatballs by 4 weeks [17]. Additionally, a study revealed that the combination of CDs and RF can mitigate fishy odor production during the storage of aquatic products. In this experiment, Zhao et al. treated crab meatballs using CDs and RF for 30 min; after 15 days of storage, trimethylamine (one of the sources of fishy odor) concentration measured at 80.95 μg/kg while total concentrations of valeraldehyde, hexanal, heptanal, and capraldehyde (another source of fishy odor) were recorded at 200.99 μg/kg—significantly lower than those observed in both HPS-treated groups and untreated controls (*p* < 0.05) [16].

Studies have demonstrated that the synergistic effect between nanoparticles and RF can effectively improve thawing quality. When RF is utilized in the defrosting and drying processes of food, its non-uniform heating is recognized as the primary limitation. Variations in temperature distribution can arise from multiple factors, such as the shape, composition, and dimensions of the food [100,106]. The high biocompatibility of magnetic nanoparticles (MNPs) enables them to disperse uniformly within the food matrix while generating heat under an alternating electromagnetic field, thereby facilitating uniform thawing. Based on this, a MNPs in combination with the RF method was developed to enhance the thawing quality of sea bass [18]. Fang et al. applied graphene nanoparticles combined with RF for thawing marine fish, with comparative evaluation against standalone RF and multiple conventional thawing methods. Their results indicated that the combined treatment moderately improved thawed fish quality while significantly accelerating the thawing process compared to traditional methods [19]. Other relevant studies not listed herein are presented in Table 1.

To assess the potential synergistic effect between the two treatments, Gutierrez’s research team employs the calculation of the Fractional Inhibition Concentration Index (FICI) [107]:

FICI = MIC_A combination_/MIC_A_ + MIC_B combination_/MIC_B_ (1)

In the formula, MIC denotes the minimum inhibitory concentration; A combination refers to the MIC of A in conjunction with B, while B combination indicates the MIC of B in conjunction with A. MIC_A_ and MIC_B_ stands for the corresponding single processing. The value of FICI ≤ 0.5, 0.5 < FICI ≤ 1, 1 < FICI ≤ 4 and FICI > 4 signify a synergistic effect, an additive effect, an indifferent effect, and antagonistic effect, respectively.

In the investigation of the integration of nanotechnology with food processing technologies, we emphasize the significance of calculating the FICI. Particularly, when more than two treatments are combined, it can facilitate a deeper understanding of the underlying mechanisms. Consequently, we advocate that researchers explore synergies through quantitative analysis.

## 4. Microwave Combined with Nanotechnology

**Figure 1:**
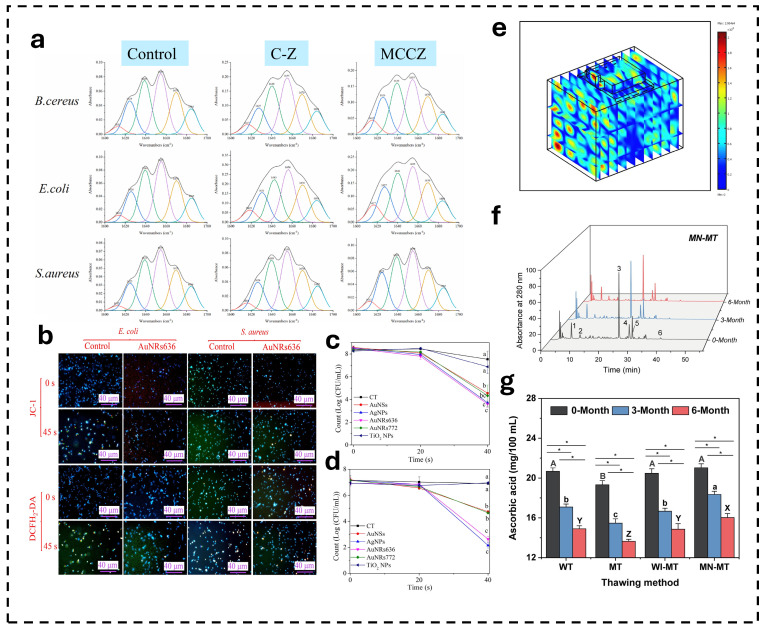
Nanotechnology is used in conjunction with MW technology. (**a**) Curve fitted results of amide I bands of three bacteria treated (Control: without any treatment; C–Z: CA–ZnO treatment for 3 h; MCCZ: CA–ZnO treatment for 3 h and MW heating for 50 s). Reprinted with permission from [23]. (**b**) The fluorescence microscopic images of *E. coli* and *S. aureus* cells subjected to AuNRs636 and MW treatment and stained with fluorescent probes (JC–1 and DCFH_2_–DA) Reproduced with permission from [24]. Inactivation of *E. coli* (**c**) and *S. aureus* (**d**) when milk samples containing AuNSs, AgNPs, AuNRs636, AuNRs772, and TiO_2_ NPs (4 µg/mL) were exposed to MW for 40 s. CT (control): bacteria suspension without NMs. Different letters indicate that the corresponding data have significant differences (*p* < 0.05). Reprinted with permission from [24]. (**e**) Example of electric fields inside a MW oven cavity across different sections. Reprinted with permission from [113]. (**f**) Chromatograms of lychee samples treated by MN–MT during 6 months storage period at 280 nm. Peak 1, catechin; peak 2, caffeic acid; peak 3, quercetin–3–*O*–rutinose–7–*O*–α–l–rhamnoside; peak 4, ferulic acid; peak 5, rutin; peak 6, quercetin. Reprinted with permission from [27]. (**g**) Changes in the content of ascorbic acid of lychee samples induced by MN−MT and other thawing treatments during storage period. Bar with different letters in same month are significantly different (*p* < 0.05). Bars with an asterisk (*) are significantly different (*p* < 0.05). Reprinted with permission from [27].

The frequency range of MW spans from 300 MHz to 300 GHz. But the heating mechanism is analogous to RF, primarily involving the rapid oscillation of dipole molecules-such as water present in food, which leads to frequent frictional interactions [108]. MW proves to be particularly effective for products with lower moisture content and irregular geometries [109,110]. Compared to traditional thermal methods, MW heating offers advantages such as rapid thermal response and minimal impact on product quality (Figure 1a).

Standalone MW treatment may exhibit insufficient microbial inactivation capacity, potentially leading to microbial hazards in food products. Liu et al. employed ZnO nanoparticles in conjunction with MW to sterilize vacuum-packed Chinese cabbage, resulting in a total colony count of less than 1 log CFU/g within 7 days and under 3 log CFU/g after 28 days—outcomes superior to those achieved through either treatment alone [22]. Furthermore, they observed that the particle size of ZnO nanoparticles diminished significantly under MW irradiation, and analogous findings have been reported in several studies [111,112]. A reduction in particle size correlates with increased reactivity due to an enhanced overall specific surface area. Zhang et al. proposed an alternative approach by modifying ZnO nanoparticles with citric acid. This modification rendered the surface lipophilic, which augmented hydrophobicity and consequently reduced nanoparticle agglomeration in aqueous environments, post-modification average particle size was approximately four times smaller. When applied for preserving animal fats, this method effectively decreased microbial counts (achieving a sterilization rate of 99.7% within 30 s) while preventing fat oxidation that leads to undesirable flavors (Figure 1c,d) [23]. Shao’s research team investigated the combined use of five different nanoparticles with MW treatment for milk sterilization. These included gold nanospheres (AuNSs), two types of gold nanorods (AuNRs636 and AuNRs772), silver nanoparticles (AgNPs), and titanium dioxide nanoparticles (TiO_2_ NPs). The numbers “636” and “772” refer to the characteristic UV absorption peaks of the two gold nanorods, which were used to distinguish between them. The results demonstrated that MW exposure not only enhanced thermal effects but also increased oxidative stress damage in bacteria. The combination of AuNRs636 (a gold nanorod type, 4 μg/mL) with MW treatment (40 s) achieved approximately 5 log CFU/mL reduction in both *E. coli* and *S. aureus* in milk. The study further highlighted the unique influence of nanoparticle morphology on MW-assisted inactivation efficacy, suggesting that varying aspect ratios may determine the outcomes of MW-nanoparticle combined treatments [24].

Akin to RF, MW heating inevitably encounters challenges related to uneven temperature distribution, due to the differential propagation of electromagnetic waves across various regions (Figure 1b) [113]. Zhu et al. reported the thawing effect achieved through a combination of MNPs, ultrasound, and MW treatment on *Dosidicus gigas*. The acoustic sensitivity of the MNPs that facilitate additional heat generation due to their high ultrasonic absorption coefficient, enabling them to convert sound energy into thermal energy [114]. The findings indicated that the treatments significantly enhanced water retention capacity, mitigated lipid and protein oxidation in *Dosidicus gigas* tissues, preserved myofibrillar protein structure integrity (Figure 1e) [26]. Xu et al. investigated the application of MNPs combined with MW treatment for fruit thawing. Compared with conventional water-immersion thawing, this approach improved thawing efficiency by 80.67% while effectively protecting thermolabile components (e.g., procyanidin B2) from degradation. Additionally, it significantly enhanced the release of phenolic compounds, particularly quercetin-3-O-rutinoside-7-O-α-L-rhamnoside [27]. Based on the mechanism of MW heating enhancement by MNPs, a study has explored their application in food industrial sludge treatment. In the reported methodology, MNPs were first uniformly incorporated into the sludge through mechanical stirring, followed by MW irradiation. The results demonstrated that this combined treatment significantly enhanced biogas production (more than three-fold increase), with the maximum biogas production rate reaching 21.2 mL/g TCOD initial/day (The biogas yield (mL) per gram of total chemical oxygen demand (TCOD) within 24 h) [31]. The improved MW heating efficiency led to elevated levels of soluble chemical oxygen demand (SCOD). This increase in SCOD subsequently provided more bioavailable compounds for fermentative microorganisms.

## 5. Ultraviolet Irradiation Combined with Nanotechnology

**Figure 2:**
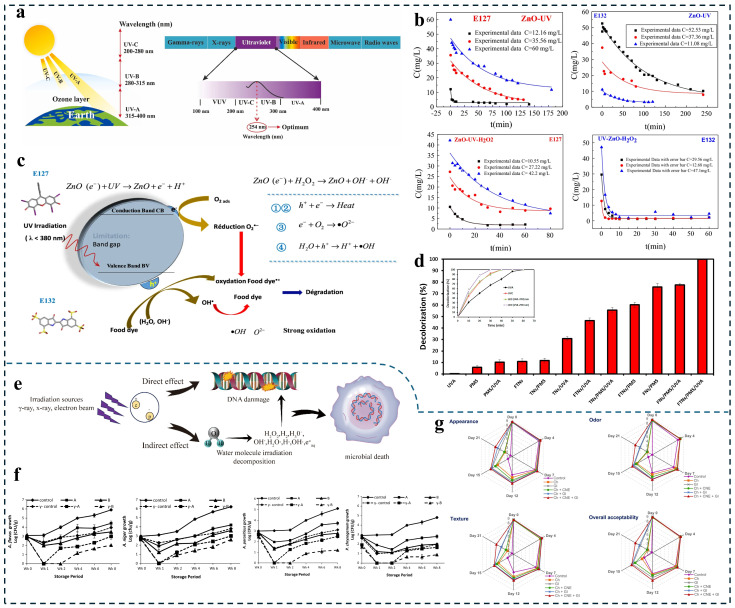
Nanotechnology is used in conjunction with UV and gamma irradiation. (**a**) The properties of the electromagnetic spectrum and UV radiation. Reprinted with permission from [115]. (**b**) The best nonlinear least–squares fit at different concentrations under two processes. Reprinted with permission from [35]. (**c**) Mechanism of degradation of food dye under ZnO nanoparticles treatment. Reprinted with permission from [35]. (**d**) The comparison of different systems for brilliant blue FCF degradation (outside) and the effect of UV sources on decolorization of brilliant blue FCF (inside). Reprinted with permission from [38]. (**e**) Mechanism of gamma irradiation. Reprinted with permission from [124]. (**f**) Fungal growth profiles of *Aspergillus flavus*, *Aspergillus niger*, *Aspergillus parasiticus* and *Penicillium chrysogenum* by treating with bioactive methylcellulose nanocomposite films for 8 weeks. Reprinted with permission from [43]. (**g**) Changes in sensory attributes of beef loins treated by synergistic processing during chilled storage. Reprinted with permission from [45].

Ultraviolet (UV) irradiation, as a food processing technology characterized by both bactericidal and non-thermal properties, occupies a frequency range between visible light and X-rays, representing an electromagnetic radiation spectrum with wavelengths spanning from 10 to 400 nm. UV is extensively employed in the processing of milk, fruits, meat products, and other items to prolong shelf life while preserving product quality (Figure 2a) [115]. The efficacy of UV is dose dependence. Increasing intensity and irradiation duration are necessary for effective microbial inactivation in food. But this escalation may adversely affect food quality since UV-sensitive components such as vitamins A and D as well as tryptophan are prone to degradation under these conditions [116].

Owing to their inherent high antimicrobial efficiency and synergistic effects when combined with UV irradiation, nano-antimicrobial materials can effectively reduce both UV intensity and exposure duration, thereby preventing potential adverse effects on food components. In 2014, Yu’s team reported utilizing ZnO nanoparticles alongside UV irradiation for food sterilization purposes. The average particle size of synthesized ZnO nanoparticles ranged from 55 to 65 nm, demonstrating inhibitory effects on microorganism growth when applied to green bean broth dishes (a total bacterial count of 2.17 log CFU/g—significantly lower than the initial count of 5.96 log CFU/g) [33]. Additively, ROS generation around ZnO nanoparticles during UV photocatalysis offers supplementary antimicrobial support [117]. Several studies have employed photocatalytic mechanisms for the degradation of mycotoxins. Raesi’s research team introduced aflatoxin B1 (AFB1) at a concentration of 10 μg/L along with ZnO nanoparticles into soymilk. Following UV irradiation for 60 min, AFB1 was completely eliminated without significantly affecting the overall acceptability of the soybean milk (*p* > 0.05) [34]. The improper disposal of food dyes utilized in daily production poses risks of environmental pollution. Ould Brahim I et al. employed ZnO nanoparticles to degrade food dyes moss red E127 and indigo carmine E132 through photocatalytic reactions under UV exposure. Their findings indicated that after 4 h of UV irradiation, degradation rates for E127 and E132 did not exceed 76%, while complete degradation of E132 occurred within 10 min when hydrogen peroxide was added as an electron acceptor (Figure 2b,c) [35].

TiO_2_, as a typical photocatalytic active material, can generate ROS under UV, demonstrates excellent killing performance against various pathogenic bacteria [118,119]. Currently, the U.S. Food and Drug Administration (FDA) has approved the use of TiO_2_ in human food and food contact materials [120]. The photocatalytic reaction of TiO_2_ under UV irradiation has been reported to be applicable in both the antibacterial process and the degradation of food dyes. LEE et al. inoculated *Salmonella typhimurium* and *E. coli* onto various vegetable leaves. Under UV light exposure, the incorporated TiO_2_ nanoparticles generated multiple ROS, resulting in microbial reductions ranging from 1.1 to 3.7 log CFU per leaf on each leaf sample [36]. Pachnowska’s team developed a synthetic Fe_3_O_4_/SiO_2_/TiO_2_ nanocomposite (NANO) designed to utilize photocatalytic reactions for yeast removal in wine, serving as an alternative preservation approach to sulfite treatment [37]. Xu et al. developed a citric acid-modified PVA film incorporating microcapsules with TiO_2_ shells that encapsulated oregano essential oil. Under UV irradiation, carvacrol-containing vapor was released. The synergistic action of UV and carvacrol disrupted bacterial cellular structures. When applied to chicken breast preservation, this approach extended the shelf life by 2 days [40]. Additionally, Zazouli et al. synthesized Fe_3_O_4_-TiO_2_ nanoparticles (FTNs) to facilitate the degradation of food dye brilliant blue FCF via photocatalytic reactions utilizing permonosulfuric acid (PMS) as an electron acceptor (this process activates conduction electrons to produce sulfate free radicals and hydroxyl free radicals [121]). The results revealed that brilliant blue FCF could be entirely degraded under UVC irradiation within 30 min (Figure 2d) [38].

Chitosan is a biodegradable, eco-friendly natural antibacterial agent. Its primary antibacterial mechanism involves electrostatic interactions that affect the structural integrity of microbial cell walls and membranes [122]. Nano-sized Chitosan possesses an increased specific surface area and enhanced antimicrobial activity [123]. Araby’s team synthesized chitosan nanoparticles and employed them in conjunction with UV for the preservation of pomegranate juice. This combined treatment demonstrated superior effectiveness over individual treatments by completely inactivating aerobic bacteria, yeast, and mold throughout a 30-day storage period while preserving a higher proportion of quality attributes, nutritional value, and health-related components [39].

## 6. Gamma Irradiation Combined with Nanotechnology

Gamma radiation is a form of ionizing radiation that can disrupt chemical bonds. When food is subjected to gamma rays, the DNA structure of microorganisms and pests becomes compromised (Figure 2e) [124,125].

As a cold procedure, it effectively preserves the texture and flavor of food; however, low doses of ionizing radiation do not ensure microbial safety while high doses may lead to oxidation and loss of vitamins in meat products—demonstrating a dose-dependent relationship [126]. Therefore, integrating nanomaterials with low-dose irradiation presents an advantageous strategy. Severino et al. developed a modified chitosan-coated citrus essential oil nanoemulsion combined with gamma radiation treatment (250 Gy) for green bean storage. On day one post-treatment, microbial counts decreased by over 3 log CFU/g and exhibited sustained antibacterial activity throughout storage duration. By day five, *Listeria* levels fell below detectable limits (<1 log CFU/g) [41]. Although transforming essential oils into nanoemulsions enhances their antibacterial efficacy, these emulsions exhibit poor stability when exposed directly to environmental conditions due to susceptibility to oxidation and cyclization. Thus, they are often encapsulated within specialized coatings [42]. Hossain’s team encapsulated oregano essential oil and thyme essential oil nanoemulsions within methylcellulose membranes, the rice following irradiation at 750 Gy for eight weeks. Compared with control samples revealed reductions in *Aspergillus flavus* and *Aspergillus parasiticus* by 2.61 log CFU/g and 3.86 log CFU/g, respectively (Figure 2f) [43]. Carvacrol and thymol from these essential oils serve as primary antibacterial agents affecting microbial cell membranes. Furthermore, membrane coating not only enhances the stability of nanoemulsion but also mitigates rapid release of active ingredients over short durations. Low-dose irradiation heightens microbial sensitivity towards environmental factors such as antimicrobials, temperature fluctuations, and pH variations which may elucidate the synergistic mechanism underlying the combination effect between nanomaterials and low-dose irradiation [127]. Severino et al. combined carvacrol nanoemulsion with gamma irradiation and modified atmosphere packaging (MAP) for pathogen inactivation on green bean surfaces. The experimental procedure involved: (1) atomizing the nanoemulsion onto green bean samples using a spray applicator, (2) packaging the samples in 3-mil nylon/EVA copolymer bags, and (3) applying irradiation treatment. The study revealed that the incorporation of MAP technology induced synergistic antimicrobial effects. Throughout the 13-day storage period, inoculated *E. coli* and *Salmonella typhimurium* populations were ultimately reduced to undetectable levels [44].

Fresh meat obtained post-slaughter typically requires freezing during transport to the point of sale. Although microbial growth is inhibited under freezing conditions, it is not eradicated. Thus, microorganisms can proliferate rapidly upon thawing due to the release of nutrients in the meat [128]. Therefore, pre-sterilization of the meat is essential. Dini et al. developed a chitosan film encapsulated with cumin essential oil nanoemulsion combined with low-dose irradiation (2.5 kGy) to prolong the shelf life of frozen beef tenderloin. Results demonstrated effective control of the proliferation of *Lactobacillus monocytogenes*, *E. coli* O157:H7, and *Salmonella typhimurium* (none detected from day 4 to the conclusion of frozen storage) (Figure 2g) [45].

## 7. Ultrasonic Wave Combined with Nanotechnology

Ultrasonic wave (UW) constitute a non-thermal, environmentally friendly food processing technology that has found extensive application in various food processes, including freeze–thawing, sterilization, and fermentation [129].

**Figure 3:**
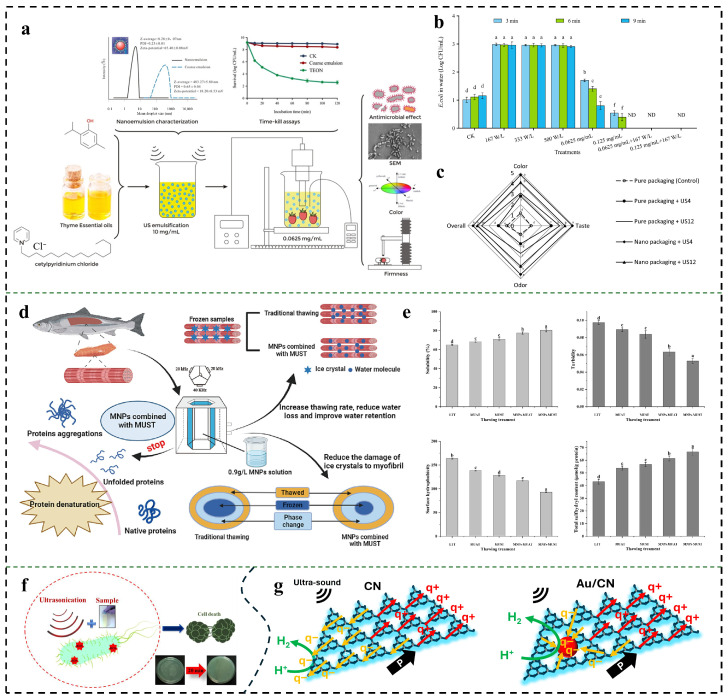
Nanotechnology is used in conjunction with UW technology. (**a**) Ultrasound can improve the decontamination effect of nanoemulsions against *E. coli* O157: H7. Reprinted with permission from [51]. (**b**) Presence of *E. coli* O157: H7 in wastewater after US, TEONs or TEON + US treatments at 3, 6 and 9 min. ND: not detected. Different letters indicate that there are significant differences between the data (*p* < 0.05). Reprinted with permission from [51]. (**c**) Effect of sonication and nanocomposite packaging on multiple sensory indicators and Overall attributes of strawberry juices. Reprinted with permission from [52]. (**d**) The possible mechanisms of MNPs combined with MUST. Reprinted with permission from [133]. (**e**) From left to right: Solubility, turbidity, surface hydrophobicity and total sulfhydryl content of salmon. Reprinted with permission from [133]. (**f**) Schematic illustrations of ROS mechanism and pathogenic bacterial degradation. Reprinted with permission from [64]. (**g**) The polarization and charge transfer process on CN and Au_2_/CN during the piezoelectric H_2_ evolution reaction. Reprinted with permission from [65].

The antibacterial properties of UW are primarily associated with its cavitation effects. In the context of food preservation and food safety control, one limitation of u UW is their inability to achieve sufficient microbial inactivation. Additionally, the cavitation effect can impact food quality to some extent, for instance by causing lipid oxidation and degradation [130]. Fortunately, studies have demonstrated that in the presence of cavitation, microorganisms exhibit increased susceptibility to antimicrobial agents, resulting in either additive or synergistic effects [50,131]. Thus, the synergistic application of antibacterial agents can effectively diminish both the duration and intensity of ultrasonic treatment. He et al. employed high-speed homogenization alongside ultrasonic homogenization to create a thyme essential oil nanoemulsion with an average droplet diameter of 8.82 nm. At a concentration of 0.0625 mg/mL combined with ultrasound treatment, *E. coli* O157:H7 inoculated on cherry tomatoes was reduced by 6.72 ± 0.02 log CFU/g at its highest—indicating synergistic antibacterial effects. Furthermore, as the concentration of the emulsion was further elevated, *E. coli* was eliminated (Figure 3a,b) [51]. Emamifar et al. integrated ZnO nanocomposite packaging with short-duration ultrasound as a dual barrier to effectively inhibit microbial growth in strawberry juice. With an ultrasonic treatment duration of 4 min, microbial proliferation in the strawberry juice was suppressed, resulting in a microbial count that remained below the spoilage threshold (6 log CFU/mL) after 14 days of storage, while also achieving a high sensory evaluation score (Figure 3c) [52]. Furthermore, researchers have investigated the combined effects of various natural essential oil nanoemulsions with ultrasound treatment across different food systems, as summarized in Table 1. Thus, the combination of brief ultrasonic exposure and nanomaterials proved to be beneficial. Additionally, studies have combined nanobubbles with ultrasound to enhance bactericidal efficacy. Researchers utilized a nanobubble generator to infuse O_2_ into water, producing oxygen nanobubbles. Subsequently, inoculated vegetable leaves were subjected to a 20 min combined treatment of nanobubbles and ultrasound in the test solution. Notably, neither treatment alone induced significant bacterial reduction, whereas the synergistic application achieved reductions of 2 log CFU/cm^2^ for *Listeria innocua* and 4 log CFU/cm^2^ for *E. coli* [60].

The integration of MNPs with ultrasound technology significantly mitigates the degradation of meat quality post-thawing. Xu et al. integrated MNPs with ultrasound for the thawing of salmon, as the MNPs enhance the efficacy of ultrasound by facilitating the conversion of acoustic energy into thermal energy [132]. The findings indicated that this approach significantly increased both the thawing rate and water retention capacity of salmon while mitigating oxidation and degradation of myofibrinogen [133]. Results and several potential mechanisms are illustrated in Figure 3d,e.

The piezoelectric effect refers to the phenomenon whereby electric charge is generated when a material exhibiting piezoelectric properties undergoes deformation due to an external force. This effect finds extensive applications in the medical field, particularly in the treatment of neurological disorders and cancer [134]. Wang’s team discovered that inert polytetrafluoroethylene (PTFE) generates ROS when subjected to ultrasonic vibrations and elucidated the underlying mechanism: within an ultrasonic field, PTFE deforms and continuously produces electric charge, thereby forming a stable electret. When exposed to periodic mechanical stimulation via ultrasound, the original charge can dissociate and subsequently react with water molecules to yield ROS [63]. The process of in situ ROS generation facilitated by ultrasound holds promise for applications such as degrading food dyes and eradicating microorganisms. For instance, Banerjee et al. developed a highly biocompatible nanocomposite composed of ZnO and chitosan that exhibited significant antimicrobial efficacy against *Enterococcus faecalis* and *E. coli* within 20 min (Figure 3f) [64]. Furthermore, Ren et al. synthesized gold-modified graphitic carbon nitride nanosheets (Au/CN), employing ultrasonic-assisted piezoelectric catalysis to degrade sugar waste from food processing while simultaneously capturing hydrogen gas produced through the reaction between H^+^ and negative charges on the surface of these nanosheets for clean fuel production (Figure 3g) [65].

## 8. Other Food Processing Technologies Combined with Nanotechnology

**Figure 4:**
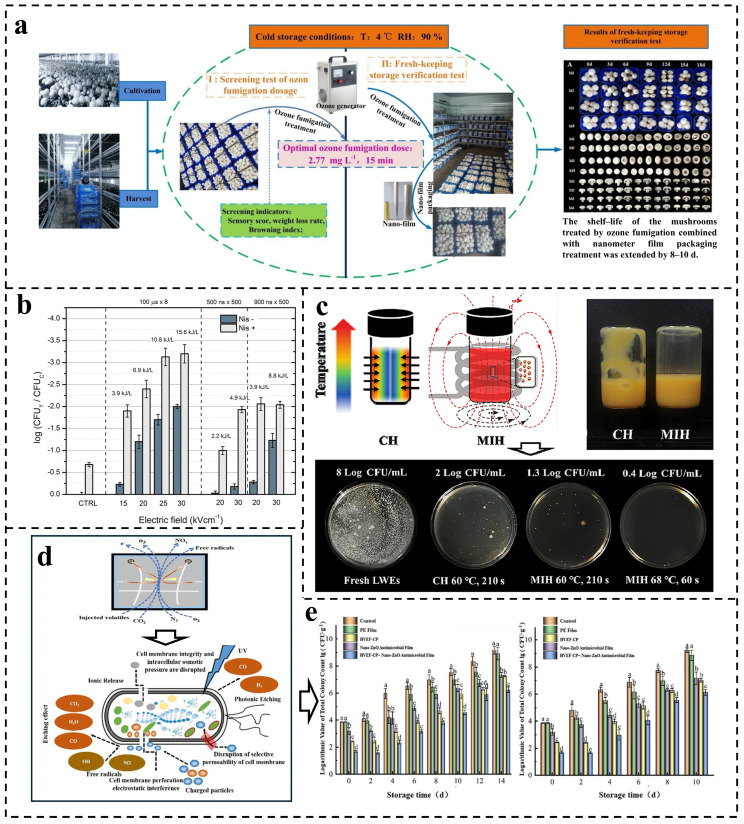
Nanotechnology is used in conjunction with other technologies. (**a**) Effects of ozone combined with nano-film on *Agaricus bisporus*. Reprinted with permission from [66]. (**b**) The bacteria CFU count and viability assay with nisin treatment for 2 h. “Nis−” corresponds to samples without nisin nanoparticles, CFU_T_ and CFU_C_ correspond to treated and control groups. Reprinted with permission from [74]. (**c**) Magnetic induction heating scheme of liquid whole egg based on magnetic microbeads. Reprinted with permission from [77]. (**d**) Plasma formation mechanism and sterilization mechanism. Reproduced with permission from [78]. (**e**) Effects of different treatments on the total colony count, grapes stored at 4 °C (**left**) and 20 °C (**right**). Different letters indicate significant differences between the data (*p* < 0.05). Reprinted with permission from [78].

In the food industry, ozone is typically generated through corona discharge or UV optical discharge methods [135] and employed for food preservation as well as degradation of fungal toxins and pesticide residues. However, a diverse array of microbial species displays varying sensitivities to ozone treatment; thus, achieving complete microbiological safety necessitates increasing the dosage of ozone used—this escalation can adversely impact food quality (e.g., reduction in vitamins and polyphenols content along with color alterations) [136]. Wang et al. reported on a polyethylene (PE) film infused with nano-silver aimed at enhancing the storage quality of *Agaricus bisporus* by mitigating its browning index (Figure 4a). Over an 18-day storage period, the combination of this nano-film with ozone (2.711 mg/m^3^) yielded superior sensory scores while effectively preserving both color and shape integrity of mushrooms, and it extended their shelf life by 6–9 days compared to conventional commercial PE films [66]. Furthermore, leveraging the photocatalytic properties of nano-TiO_2_, studies have indicated its integration with ozone oxidation technology for the effective degradation of volatile organic compounds (VOCs) present in cooking fumes. Li et al. developed a continuous flow reaction system specifically designed for VOCs, which comprises a photocatalytic device (a glass fiber filter coated with a multilayer nano-TiO_2_ film) and an ozone oxidation reactor. When the VOCs go through the reactor, the degradation efficiency can reach up to 94% [67].

Several studies have employed microbubble nanotechnology to generate ozone micro-nano bubbles (MNBs). This technique effectively reduces the diameter of ozone bubbles, enhances their mass transfer coefficient, and increases the solubility of ozone in aqueous systems. Additionally, due to the augmented specific surface area, ozone MNBs can remain stable for extended periods [137], which is crucial for their efficacy in practical applications. Shi et al. integrated an ozone generator with a MNBs generator to produce ozone bubbles ranging from 5 nm to 20 μm in diameter. They subsequently utilized MNBs for maintaining the quality of parsley post-harvest, setting the concentration at 2.5 mg/L and treatment duration at 10 min. Their findings indicated that parsley treated with ozone MNBs exhibited enhanced nutritional qualities while preserving sensory attributes [69]. Lin et al. developed a novel piezocatalytic sterilization system, integrating spontaneously polarized ceramics (SPC) with O_3_ MNBs based on ozone nanobubble technology. The collapse process of O_3_ MNBs induces piezoelectric effects in SPC, generating a strong polarized electric field. When O_3_ adsorbs onto SPC surfaces, it decomposes into •OH. This system achieves fruit preservation through the electroporation of bacterial membranes by the polarized electric field and potent oxidation by •OH. The system demonstrated remarkable antimicrobial efficacy, inactivating 7 log CFU/mL of both *E. coli* and *S. aureus* within 20 min, while significantly enhancing the preservation quality of Kyoho grapes [70].

PEF is recognized in the food industry as a viable alternative to conventional thermal sterilization methods. When exposed to PEF, the structural integrity of harmful microorganisms’ cell membranes in food is compromised, leading to their inactivation. It is important to note that microbial resistance or sublethal states may arise depending on factors such as electric field intensity, microbial growth conditions, and species variations. In the sublethal state, the destruction of microbial structure may be reversible, which will affect the quality of food [138,139]. Recent studies have explored the synergistic effects of combining nanoparticles with PEF to enhance microorganism inactivation efficacy. Upon application of PEF, microorganisms experience membrane perforation that allows nanoparticles to penetrate the cellular interior through these pores for effective inactivation [140]. This combination not only enhances antibacterial activity but also mitigates the development of microbial resistance and potential restoration of structure during sublethal states. For instance, Carvalho et al. developed a nanoemulsion containing peppermint essential oil which effectively inactivated *E. coli* O157:H7 present in guava juice and mango juice when used alongside PEF treatment. The findings indicated that the MIC of the essential oil nanoemulsion was 5 μL/mL, with a reduction in bacterial concentration by 5 log CFU/mL (initially at 7 log CFU/mL) observed in guava juice and mango juice after durations of 70 and 90 min, respectively. Notably, when integrated with a PEF (150 μs, 20–30 kV/cm), a concentration of 0.16 μL/mL can attain an equivalent sterilization effect, demonstrating a synergistic interaction between the two [73]. Nisin has been established as an effective antibacterial agent primarily due to its interaction with phospholipid components within microbial cell membranes. Novickij et al., loaded nisin onto pectin nanoparticles aimed at enhancing nisin’s environmental stability before integrating it with nanosecond PEF for investigating *E. coli* inactivity dynamics. The results revealed that pectin-coated nisin (14.9 μM) exhibited lower MIC values compared to free nisin (29.8 μM), showcasing enhanced synergistic antibacterial effects under PEF operating at durations between 500–900 ns and intensities around 30 kV/cm—thereby improving *E. coli* inactivity dynamics (Figure 4b) [74].

Research indicates that exposure to high-intensity magnetic field (MF) alters the permeability of microbial cell membranes, thereby disrupting cellular homeostasis [141]. Novickij et al. proposed a novel approach to PEF integrated with nanotechnology, developed MNPs loaded with nisin. In an alternating magnetic field (AMF), these nanoparticles exhibited not only bactericidal activity but also generated a thermal effect. Specifically, at a MF intensity of 125 mT, *Listeria* levels could decrease by 3 logs within 30 min while significant membrane damage is observed in the bacteria [76]. Magnetically responsive micro-nanomaterials produce heat when subjected to an AMF. Wang et al. designed a magnetic microbead that utilizes magnetic induction for pasteurizing liquid whole eggs as an alternative to conventional thermal convection methods. Notably, at 68 °C for 60 s, *Salmonella enteritidis* counts were reduced by 7.6 log without compromising the sensory quality of the whole egg due to more uniform heating (Figure 4c) [77].

CP is an ionized gas consisting of various charged particles at low or ambient pressure, including excited-state atoms, ions, and photons [142]. It is primarily generated through corona discharge and dielectric barrier discharge methods in the food industry, and utilized for food preservation, seed germination, and the degradation of toxic substances [143]. Li et al. proposed the synergistic use of a ZnO nanoparticles antibacterial film combined with CP for grape preservation. Compared to using either CP or antibacterial films alone, this combination significantly enhanced the antibacterial efficacy (99.34%) and extended the shelf life of grapes by 6–8 days under refrigeration conditions (Figure 4d,e) [78]. Amini’s research team fabricated a cellulose/PVA/nanoclay (CPN) nanocomposite and combined it with CP treatment to ensure microbial safety during wheat flour storage. The experimental group treated with CP followed by CPN packaging demonstrated superior performance compared to individual treatments. Throughout the 90-day storage period, the viable count of *Aspergillus flavus* inoculated wheat flour was reduced from 4.2 log CFU/mL to undetectable levels and preserved the gluten and starch content of wheat flour throughout the storage period [80]. Cid R. González-González et al. developed linalool-loaded nanoemulsions and investigated their combined effect with CP on pathogenic bacteria inactivation in ready-to-eat chicken. The study revealed that CP-pretreated bacterial cells exhibited enhanced susceptibility to antimicrobial nanoemulsions, which may be attributed to CP-induced mechanical damage to cellular membranes resulting in pore formation [82].

## 9. Safety and Regulatory Road

While nanotechnology is often widely regarded as having the potential to address “global challenges,” critical translation barriers remain in its journey from the laboratory to industry. Preliminary cytotoxicity screenings are frequently employed to assess biosafety, yet truly comprehensive biocompatibility evaluations require more in-depth investigation [144]. Specifically, we believe that considerations and actions can be taken from the following aspects.

### 9.1. Possible Exposure Pathways

Human exposure to engineered nanomaterials (ENMs) in food occurs primarily through two pathways: direct ingestion of food containing nanomaterials, and indirect migration of nanomaterials into food (e.g., when ENMs serve as active components in food packaging). Direct exposure includes the consumption of foods containing nano-additives, such as TiO_2_ (as a colorant), SiO_2_ (as an anti-caking agent), and nano-encapsulated nutrients or flavors. Indirect exposure arises from the transfer of nanoparticles—such as nano-silver, nanoclay, ZnO, and TiO_2_—from packaging materials into food. This migration is influenced by factors including nanoparticle properties, polymer matrix, contact time, temperature, and food composition (e.g., acidity, fat content). Studies have reported detectable migration of Ag, Si, and Ti ions into food simulants, although typically below current regulatory limits [145,146]. Occupational exposure during the manufacturing and handling of nano-enabled food packaging also presents potential risks via inhalation and dermal contact.

### 9.2. Transformation During Processing and Storage

ENMs may undergo physical or chemical transformations during food processing, storage, and human digestion, altering their bioavailability and toxicity [147]. Processing steps such as heating, high-pressure treatment, or UV irradiation can induce nanoparticle aggregation, dissolution, or surface modification. During storage, interactions between nanoparticles and food components (e.g., proteins, lipids, organic acids) may affect their stability and migration behavior. Most existing studies have been conducted in food simulants, which still leaves a significant gap in understanding the complex transformation and migration processes of nanoparticles under real production conditions.

### 9.3. Toxicological Indicators and Research Considerations

The toxicity of nanoparticles can be triggered through various mechanisms depending on their composition and morphology. For instance, inorganic nanoparticles tend to generate ROS, such as superoxide, singlet oxygen, hydroxyl radicals, and hydrogen peroxide—which constitute one of the most critical factors underlying their toxicity [148]. Moreover, sensitivity to nanotoxicity varies across different parts of the human body. Organs such as the liver, lymph nodes, and spleen absorb nanomaterials at a significantly faster rate than others, leading to potential accumulation that may provoke inflammatory responses and DNA damage.

Future research on the application of nanomaterials in food must therefore consider the following aspects: realistic exposure doses and chronic exposure scenarios, including assessments of cytotoxicity, oxidative stress, genotoxicity, and organ-specific accumulation; the use of relevant food models to simulate migration and digestive processes; and dedicated safety evaluation for susceptible subpopulations, such as children and immunocompromised individuals.

### 9.4. Regulatory Requirements and Future Directions

Globally, the regulation of nano-enabled foods and packaging materials varies significantly, reflecting different approaches to risk assessment [149]. In the United States, the FDA manages these materials under existing frameworks for food additives and food contact materials, generally adhering to the principle of “substantial equivalence” unless new safety concerns arise. In contrast, the European Union regulates novel nano-food contact materials under Regulation No 1935/2004 and relevant European Food Safety Authority (EFSA) guidelines, which require pre-market authorization and the submission of comprehensive data, including physicochemical characterization, migration studies, and toxicological evidence [147].

Key regulatory challenges currently include: the lack of standardized methods for detecting and characterizing ENMs in complex food matrices; uncertainties regarding long-term, low-dose exposure effects; the need to employ computational modeling and tiered testing strategies to reduce animal testing; and the necessity of harmonizing definitions and risk assessment procedures internationally. Looking ahead, adopting a proactive “safety-by-design” strategy, implementing continuous monitoring of nano-food products, and promoting transparent labeling where appropriate will be crucial for balancing technological innovation with public health protection. Enhanced collaboration among academia, industry, and regulatory bodies is essential to establish a robust, adaptable, and forward-looking safety and regulatory framework, thereby paving the way for the responsible application of nanotechnology in the food sector.

## 10. Future Perspectives

This paper reviews representative cases of integrating nanotechnology with food processing techniques, serving as a modular optimization approach for the food industry. The combination of nanomaterials and food processing technologies demonstrates enhanced bactericidal effects and improved food quality preservation. These enhanced effects originate from synergistic or additive interactions between nanomaterials and food processing technologies. A representative synergistic mechanism involves processing-induced mechanical damage to microorganisms, which increases their susceptibility to antimicrobial agents. Additionally, the direct incorporation of nanoparticles can simplify production processes, thereby reducing potential food contamination risks. The multifunctional applications of nanomaterials are significantly enhanced by their diverse mechanisms of action, particularly in improving food safety through reducing the potential development of pest and microbial resistance. In summary, the integration of nanomaterials with food processing technologies exhibits significant potential for applications in food safety, preservation, quality, and green processing [150].

However, the toxicity of nanoparticles remains largely unexplored, and such toxicity could vary based on several factors such as particle type, concentration, and individual sensitivity. Certain metallic nanoparticles might possess potential toxicity, posing risks to human organs [91]. These potential safety concerns restrict the progress of nanotechnology within the food industry.

In conclusion, the numerous positive outcomes from the application of nanotechnology demonstrate its potential to enhance food processing, offering viable solutions for the development of safe, efficient, and sustainable food supply systems. Moving forward, it is imperative to develop greener and safer nanomaterials while exploring novel applications of nanotechnology in food processing. Concurrently, it is essential to establish effective predictive models to assess the toxicity of nanomaterials, as well as to define their application scope and permissible limits within the food processing industry.

## Figures and Tables

**Table 1:** The application and primary mechanisms of nano-assisted processing technology in food processing.

Food Processing Technology	Material ^a^	Sample ^b^	Category ^c^	Method ^d^	Dosage ^e^	Main Findings ^f^	Limitation ^g^	Ref.
Radio frequency (RF)	ZnO nanoparticles 80–100 nm	Carrots	Food preservation	Immerse the carrots in the ZnO nanoparticle suspension	Nano ZnO: 0.04 g/kg RF: 6 kW, 27 MHz, board spacing: 20 mm, time: 20 min	Achieved <3 log CFU/g; 60-day shelf life; Best quality: minimal color change, maintained hardness, low carotenoid loss	The application in diverse food matrices still needs to be evaluated	[14]
	CDs ≤1 nm	Boiled gansi dish	Food safety	Add CDs directly to gansi dish	CDs: 0.24 mg/mL, RF: board spacing: 120 mm, sample center temperature: ~87 °C	Under optimal conditions, inoculated *Bacillus subtilis* was reduced by 3.62 log CFU/mL; The samples maintained good texture, flavor, and appearance, achieving a higher sensory score	The physical and chemical stability as well as the antibacterial activity retention of CDs during long-term storage have not been fully investigated	[15]
	CDs 0.54–1.50 nm	Crab meatballs	Food quality	Add CDs directly to crab meatballs	CDs: 0.2 mg/g RF: board spacing: 20 mm, sample center temperature: ~67.49 °C	Under optimal conditions, inoculated *Bacillus subtilis* was reduced by 2.25 log CFU/mL; Shelf life was extended by at least 15 days; joint treatment effectively inhibited lipid oxidation and reduced aldehyde substances linked to fishy odor	The physical and chemical stability as well as the antibacterial activity retention of CDs during long-term storage have not been fully investigated	[16]
	CDs ≤10 nm	Fried meatballs	Food preservation	Package meatballs by CDs/PVA film	CDs: 0.5% (*w*/*w*) RF: 6 kW, 27 MHz, board spacing: 20 mm, time: 20 min	The combined treatment group had a total colony count of 3.72 log CFU/g in week 6, significantly lower than the single treatment group; Its shelf life was extended by 4 weeks versus the control; It effectively delayed both lipid oxidation and protein decomposition	The preparation of the packaging film is complex, and the cost for large-scale application needs to be taken into consideration	[17]
	MNPs	Sea bass	Food quality	Immerse the sea bass filets completely in the MNPs suspension	MNPs: 0.1 mg/mL RF: 40.68 MHz, 400 W	Improved the thawing quality of fish meat, including the texture, protein cross-linking network and water retention ability	It may not be applicable to the types of foods those with high fat content or loose structure	[18]
	Graphene nanoparticles	Hairtail	Food quality	Immerse the hairtail in graphene nanoparticles suspension	Nanoparticle: 0.1 mg/mL RF: 40.68 MHz	The samples in the combined treatment group showed the highest whiteness and brightness, and had less thawing loss	This scheme may have different levels of applicability for various types, shapes or sizes of food	[19]
	Starch-based nanocomposite 62 nm	Pepper	Food preservation	Package pepper by starch-based nanocomposite film	RF: 23.73 MHz, 2 min	Under optimal conditions, the reduction amounts of fungi and bacteria were 2 log CFU/g and 3 log CFU/g, respectively; Samples were stored at 6 °C for 30 days	The mechanical properties and barrier properties of the composite film may change during long-term storage	[20]
	Cinnamon essential oil nanoemulsion ~80.28 nm	Pork	Food safety	Add cinnamon essential oil directly to samples and mix	Nanomaterial: 0.125% nanoemulsion + 0.25% ε-PL RF: freezing point temperature: 71 °C, time: 13.5 min	The combined treatment reduced *Salmonella* by up to 8.06 log CFU/g; The shelf life was 12 days under refrigeration conditions; The combined treatment had a slight effect on the odor, but significantly improves the texture characteristics (hardness, elasticity, and chewiness)	The research does not cover other meats or food systems	[21]
Microwave (MW)	ZnO nanoparticles 30–40 nm	Chinese cabbage	Food preservation	Immerse cabbage in ZnO suspension	ZnO: 0.02 g/kg MW: 2450 MHz, 400 W, 150 s	The combined treatment group resulted in a total colony count of less than 1 log CFU/g within 7 days, and 2.54 log CFU/g after 28 days; The shelf life had been extended to 28 days; Better color and texture	Not involving any other vegetables or food systems	[22]
	Citric acid modified ZnO nanoparticles ~394.2 nm	Animal fat (poultry fat mixed with lard)	Food preservation	Add nanoparticles directly to the animal fat at	CA-ZnO: 0.05 g/kg MW: 2450 MHz, 700 W, 90 s	The combined treatment reduced the total colony count by 3.5 log CFU/g; The combined treatment had a lower adverse effect on the flavor and a lower degree of fat oxidation	For animal fats, their MW absorption efficiency is lower than that of water-based systems, which may affect the efficiency of sterilization	[23]
	AuNSs: ~12.63 nm AuNRs636: ~13 nm AuNRs772: ~19.55 nm AgNPs: ~43.63 nm TiO_2_ NPs: ~120nm	Milk	Food safety	Add nanomaterials directly to milk and mix	AuNRs636: 4 μg/mL MW: 2.45 GHz, 1000 W, 40 s	AuNRs636 + MW achieved a reduction of approximately 5 log CFU/mL in *E. coli* and *S. aureus*	No evaluation was made of the sensory properties of the sample	[24]
	Polysaccharide- and protein-based nanocomposite antibacterial films 100–240 nm	Meat	Food preservation	Package meat by antibacterial films	Nanoparticle: 2.5% (*w*/*w*) MW: 3 W/cm^2^, 6 min	The sterilization rates of the combined treatment for *E. coli* and *S. aureus* were 98.6% and 97.2%, respectively; The shelf life was extended to 5 days; Lipid oxidation slowed down, water-holding capacity increased, and appearance remained better	It faces challenges in large-scale continuous production	[25]
	MNPs 20–50 nm	*Dosidicus gigas*	Food quality	Immerse *Dosidicus gigas* in MNPs suspension	MNPs: 0.9 mg/mL MW: 2450 MHz, 300 W	The water-holding capacity of the combined treatment group significantly increased, with the lowest degree of oxidation and better preservation of protein structure	The dispersion stability and aggregation properties of MNPs when used for long-term circulation in the thawing solution are issues	[26]
	MNPs 20 nm	Lychee	Food quality	Immerse lychee in MNPs suspension	MNPs: 0.1 mg/mL MW: 2450 MHz, 300 W	The combined treatment had a higher thawing efficiency, effectively protected phenolic substances and vitamin C, maintained better color, and kept the pH value, total acidity, soluble solids and other indicators stable	The soaking treatment is not suitable for large-scale, continuous industrial production lines	[27]
	MNPs 10–30 nm	Largemouth bass	Food quality	Immerse largemouth bass in MNPs suspension	MNPs: 0.1 mg/mL	The samples subjected to combined treatment performed the best in terms of elasticity and chewability, and had the least water loss	The issue of recycling of nanomaterials has not been discussed	[28]
	MNPs 20–50 nm	Atlantic salmon	Food quality	Immerse Atlantic salmon in MNPs suspension	MNPs: 0.9 mg/mL MW: 500 W	MNPs-MW thawing best preserved salmon myofibrillar protein stability during freeze–thaw; It formed smaller, rounder ice crystals, minimizing cellular damage; This improved overall preservation quality	MNPs’ long-term safety for consumption still needs further verification	[29]
	Nano silver 65.34 nm	Sea cucumber	Food quality	Immerse in the nano-silver solution to form a coating	Nano silver: 0.045 mg/mL	The samples processed in a combined manner had better rehydration properties, and there were no significant differences in terms of color, appearance, texture, and flavor compared to the traditional method	The synergistic effect is not obvious	[30]
	MNPs ~109 nm	Food industrial sludge	Green processing	Add MNPs directly to sample	MNPs: 5 mg/100 mL MW: 2.5 W/mL, 3 min	Combining MW with MNPs boosted sludge biogas production and enabled dielectric monitoring of pretreatment efficacy	The composition of the sludge may affect the uniformity of the distribution of MNPs and the uniformity of the microwave thermal effect	[31]
	MNPs 50 nm	*Pistacia vera* L.	/	Immerse *Pistacia vera* L. in MNPs suspension	MNPs: 0.2 g/50 g MW: 400 W, 120 min	Amine-functionalized magnetite nanoparticles enhanced MW-assisted extraction efficiency, particularly for essential oils from *Pistacia vera* hulls	The issue of residual and migration of nanomaterials in food was not discussed	[32]
Ultraviolet (UV)	ZnO nanoparticles 55–65 nm	Green bean broth dishes	Food safety	Add ZnO directly to sample	ZnO: 0.04 g/kg UV: 196.98 V and 49.79 min	Inhibited the growth of bacteria and fungus, the total amount was 3.69 and 1.87 CFU/g, respectively, in 16 days; Maintains texture, color, nutrition	No specific extension of the storage time was provided; Nano ZnO has undergone aggregation	[33]
	ZnO nanoparticles	Soybean milk	Food safety	Add ZnO directly to sample; 0.1 mg/mL;/V and 60 min	ZnO: 0.1 mg/mL UV: 60 min	100% complete removal of AFB1 from the aqueous solution was achieved; Soymilk did not experience a significant impact on its overall acceptability	Other components of the complex food matrix may have an impact on the photocatalytic results	[34]
	ZnO nanoparticles	/	Green processing	Add ZnO directly to solution	ZnO: 2 g/L UV: 4 h	For dye E132, complete degradation could be achieved within 10 min	Other components of the complex food matrix may have an impact on the photocatalytic results	[35]
	Nano TiO_2_ 21 nm	Vegetables	Food safety	Spray the TiO_2_ suspension onto the surface of the leaves	Nano TiO_2_: 1.50 μg/mL UV: 6000 J/m^2^	For internalized *Salmonella* and *E. coli*, a maximum reduction of 3.7 log CFU per leaf was achieved	The variety of vegetables may lead to differences in the sterilization effect	[36]
	Fe_3_O_4_/SiO_2_/TiO_2_ nanocomposite 70–90 nm	Wine	Food preservation	Add nanocomposite directly to solution; 500 mg/L;/V and 30 min	Nano: 500 mg/L UV: 30 min	Could effectively reduce the viable cell count of *Saccharomyces cerevisiae* (~5 log CFU/mL); Positive regulation of UV-C on the polyphenol components of wine maintained chemical stability	The costs in large-scale production, as well as the color and texture of the wine may affect the penetration rate of UV-C	[37]
	Fe_3_O_4_-TiO_2_ nanoparticles 40–70 nm	/	Green processing	Add nanocomposite directly to solution	Nano: 0.8 g/L UV: 2.52 mW/cm^2^, 60 min	100% degradation of the food dye bright blue FCF was achieved	The low ion leaching rate may imply that the need for further discussion regarding a broader range of applications is imminent	[38]
	Chitosan nanoparticles 43.2–114 nm	Pomegranate juice	Food preservation	Add nanocomposite directly to juice	Chitosan: 175 μL/mL UV: 0.356 J/cm^2^ and 30 min	The pathogenic bacteria inoculated were completely inactivated in the pomegranate juice. Extended the shelf life to 30 days; Effectively retained the health-related components in the pomegranate juice	The complex components of the juice may affect the activity of the nanoparticles	[39]
	Modified PVA films loaded with oregano essential oil microcapsules ~102 nm	Chicken breasts	Food preservation	TiO_2_ is incorporated into the modified PVA film for food packaging	/	It shows antibacterial effects against *E. coli* and *S. aureus*; it can extend the shelf life of chicken breast by at least 2 days	During the storage period of the film, the encapsulation stability of the microcapsules and the dispersion stability of the nanomaterials are the key factors	[40]
Gamma irradiation	A modified Chitosan-coated citrus essential oil nanoemulsion ~176 nm	Green beans	Food preservation	Apply the solution onto the surface of the green beans to create an active coating	Nanoemulsion: 0.05% (*w*/*v*) Gamma: 0.25 kGy	The microbial count decreased by 3 log CFU/g in 1 day, and 4 days later, it was below the detection limit at 50 CFU/g; Maintained food quality for 12 days; It had no adverse effect on the color, but it will caused the hardness of the sample to decrease	The adhesion, uniformity and antibacterial effect of the coating may vary on different types and surface forms of vegetables	[41]
	Microspheres of alginate encapsulating thyme essential oil ≤200 nm	Pork	Food preservation	Microbeads were applied onto the pork	Microbeads: 15 g/25 g Gamma: 3 kGy	Mesophilic total flora was 2 log CFU/g after 14 days; The shelf life had been extended by 12 days	The stability of the microbeads under irradiation treatment has not been evaluated	[42]
	Methylcellulose-based membranes incorporating nanoemulsions of oregano and thyme essential oils ≤100 nm	Rice	Food safety	Integrate the nanomaterials into the film	Gamma: 0.75 kGy	The fungal growth decreased by 2 log CFU/g within 8 weeks; The shelf life was extended to 8 weeks	When the film is used on the surface of foods with different humidity and fat content, its water vapor barrier property and the rate of essential oil release may be affected	[43]
	Modified chitosan-based coating containing nanoemulsion of essential oils ≤200 nm	Green beans	Food safety and preservation	Apply the solution containing nanoemulsions onto the green beans	Mixture: 0.05% (*w*/*v*); Gamma: 0.25 kGy	Reduced *E. coli* and *Salmonella* to undetectable levels; Extended the shelf life to 13 days	The complexity and cost of gas-chilled packaging and irradiation facilities may be highly industrialized	[44]
	Cumin essential oil nanoemulsion coated with Chitosan film 89.61 nm	Beef tenderloin	Food preservation	Packaging food with a composite film containing nanomaterials	Chitosan and nanoemulsions: 1% Gamma: 2.5 kGy	Reduced the microbial concentration from 7 log CFU/g to undetectable levels; Extended the shelf life to 21 days; Maintained a good sensory score throughout the 21-day storage period	The long-term stability of nanoemulsions has not been verified in actual food packaging	[45]
	Bioactive polyethylene film 3–35 nm	Strawberry	Food preservation	Prepared nano-composite membrane is used for packaging strawberries	Active formula: 300 μL/2 g, CNCs 0.375%, glycerol 0.625% Gamma: 0.5 kGy	The inhibition rate against the four types of microorganisms was the highest at 84%; The shelf life was extended to 12 days; The total phenol and anthocyanin contents significantly increased after 12 days of storage	The study did not extend to other fruits with high water content or high acidity	[46]
	Chitosan/essential oils/silver nanoparticles composite film AgNPs: ~62.7 nm	Strawberry	Food preservation	Prepared antibacterial compo-site membrane is used for packaging strawberries	Gamma: 1 kGy	The *Listeria* was completely inhibited, and the *E. coli* was reduced by 3.4 log CFU/g; The shelf life was extended to 12 days; Effectively maintained sensory quality	The release of AgNPs needs to be further evaluated	[47]
	Alginate-cellulose nanocrystal microcapsules encapsulating essential oils	Dry fermented sausages	Food preservation	Add microcapsulated essential oil to the dry-fermented sausages; 1.5 kGy	Gamma: 1.5 kGy	Reduced the quantities of multiple microorganisms to undetectable levels; The shelf life was extended to 8 weeks; Protected the color of the sausage	The structural integrity and the release behavior of active components of microcapsules during long-term storage need to be further verified	[48]
	Microencapsulated oregano essential oil, cinnamon essential oil, and nisin	Ham	Food preservation	Apply the antibacterial agent in the form of microcapsules onto the surface of the ham	Gamma: 1.5 kGy	*Listeria* was undetectable (≤50 CFU/g) on the first day of storage; The safe storage period of ham was extended to over 28 days at a temperature of 4 °C	The process from preparation to coating is complicated	[49]
Ultrasonic wave (UW)	Oregano and thyme oil nanoemulsion 35–133 nm	Lettuce	Food safety	Soak the chopped lettuce in the essential oil solution	Nanoemulsion: ≥0.018% (*v*/*v*) UW: 26 kHz, 200 W and 5 min; Pulse mode, 2 s on/2 s off	Salmonella levels had decreased to below the detection limit	No assessment was made of the changes in sensory properties	[50]
	Thyme essential oil nanoemulsion 8.28 nm	Cherry tomatoes	Food safety	Soak the cherry tomatoes in the essential oil solution	Nanoemulsion: 0.125 mg/mL UW: 20 kHz, 167 W/L and 3 min; Pulse mode, 2 s on/2 s off	The number of microorganisms on the surface of the cherry decreased by 6.72 log CFU/g; There were no significant changes in color or hardness, and the color tone was improved to some extent	The application in more foods needs to be evaluated	[51]
	ZnO nanoparticles composite antibacterial packaging 10–30 nm	Strawberry juice	Food preservation	Put the strawberry juice into the packaging bag which contains nano ZnO as the main antibacterial ingredient	ZnO: 3: 100 (*w*/*w*) UW: 24 kHz, 400 W and 12 min; Continuous mode	Aerobic bacteria and mold yeast counts remained below the spoilage threshold (6 log CFU/mL) in 35 days, significantly outperforming the control; Shelf life was extended to 35 days; The combined treatment group achieved the highest sensory score	The migration effect of nano ZnO into strawberry juice has not been evaluated	[52]
	Basil essential oil nanoemulsion 198–228 nm	Sprouts	Food safety	Soak the sprouts in a solution containing nanoemulsion	Nanoemulsion: 0.5 mg/mL UW: 20 kHz, 241 W/cm^2^ and 10 min; Pulse mode, 5 s on/5 s off	The reduction in *Salmonella* on the surface of bean sprouts was 4.44–5.00 log CFU/g; The color of the sprouts had slightly darkened, and their hardness had increased	The application in more foods needs to be evaluated	[53]
	Oregano essential oil nanoemulsion ~132.43 nm	Blueberry	Food preservation	Immerse the blueberries in a cleaning system containing nano emulsion	Nanoemulsion: 0.06 μL/mL UW: 255 W/cm^2^, 10 min; Pulse mode, 2 s on/2 s off	The *Listeria* count decreased by 6.35 log CFU/mL, and reached 100% after 9 min; The shelf life was extended to 14 days; Blueberries showed no significant differences from the control group in terms of color, hardness, and antioxidant activity	The application in more foods needs to be evaluated, especially for complex food matrices	[54]
	Carvacrol nanoemulsion 149 nm	Cabbage	Food preservation	Soak the cabbage leaves inoculated with bacteria in a system containing nanoemulsion	Nanoemulsion: 0.3 mg/mL UW: 241 W/cm^2^, 10 min; Continuous mode	The *Listeria* count decreased by 2.039 log CFU/g; The shelf life was extended to 4 days; Significantly reduced the weight loss rate of cabbage, with no significant differences in color and hardness.	The surface structures of different vegetables (such as waxy layers and stomata) may affect the adhesion, penetration and sterilization efficiency of nanoemulsions	[55]
	Cinnamon leaf oil emulsion containing cationic surfactants ~350 nm	Fresh-cut endive	Food safety	Soak the fresh-cut endive in the cleaning solution containing the nanoemulsion;	UW: 40 kHz, 140 W and 3 min; Continuous mode	*Listeria* and *E. coli* O157:H7 decreased by 1.58 and 1.60 log CFU/g, respectively; The shelf life was extended to 8 days; The degree of browning was significantly lower than that of the control group and the water washing group, and it had no significant effect on hardness and total phenolic content	The long-term stability of nanoemulsion has not been evaluated	[56]
	Thyme essential oils nanoemulsion	/	Food safety	The nanoemulsion was directly added to the bacterial suspension	Nanoemulsion: 0.375 mg/mL UW: 20 kHz, 255 W/cm^2^ and 9 min; Continuous mode	The suspension of *E. coli* O157:H7 was reduced by 7.42 ± 0.27 log CFU/mL	Not applied in the actual sample	[57]
	Thymus vulgaris essential oil nanocapsules 84.26 nm	Green bell peppers	Food preservation	Soak the sweet peppers in the nanolotion of thyme essential oil	Nanocapsules: 500 mg/L UW: 100 W/cm^2^ and 10 min; Continuous mode	Combined treatment significantly reduced the total number of viable bacteria, as well as the counts of molds and yeasts; The shelf life has been extended to 10 days; The combined treatment group had the best color and appearance, while the total phenolic content and antioxidant activity were maintained	The weight of green peppers has decreased	[58]
	Zanthoxylum schinifolium essential oil nanoemulsions	Fresh-cut cucumber	Food safety	Soak fresh-cut cucumbers in the nanoemulsions	Nanoemulsion: 1 mg/mL UW: 230 W/cm^2^ and 12 min; Continuous mode	Reduced *E. coli* O157:H7 by 8.91 log CFU/mL; Stored at 4 °C for 12 days; The physical indicators such as color, hardness and weight loss remained better	The molecular mechanism of the synergistic effect remains to be evaluated	[59]
	O_2_ nanobubbles	Spinach	Food safety	Immerse the spinach leaves in the O_2_ nanobubbles solution	/	Combined treatment can achieve a removal rate of 2 log CFU/cm^2^ for *Listeria* and 4 log CFU/cm^2^ for *E. coli*	Lack of discussion on the coordination mechanism; No assessment was made regarding the sensory properties	[60]
	Hydrophobic natural deep eutectic solvents nanobubbles 237.36 nm	Walnut shell	/	Dissolve the nanobubbles in a hydrophobic solvent, then mix them with the walnut shell powder and conduct ultrasonic-assisted extraction	UW: 280 W and 46 min; Continuous mode	Compared with the initial ethanol extraction, the extraction rate of triterpenic acids had significantly increased	The size of the nanobubbles may affect their diffusion and interface interaction	[61]
	Chitosan nanocomposite water-retaining agent 164.2–615.1 nm	Frozen crayfish	Food quality	Soak the crayfish in the nanocomposite	UW: 60 W and 1 min; Continuous mode	The combined treatment group had the lowest thawing loss (9.58%), the highest moisture content (65.32%), the lowest	The long-term structural stability of the water-retaining agent needs to be further evaluated	[62]
	Polytetrafluoroethylene 1–5 µm	/	Food safety and green processing	/	/	Within 60 min, the removal rate of methyl orange dye reached 89.7 ± 2.9%. In the simulated water disinfection experiment, the inactivation rate of *E. coli* reached 99.7%	For large-scale food or water treatment applications, the energy efficiency and economic aspects need to be further evaluated	[63]
	ZnO/chitosan bio-nanocomposite 30.13 nm	/	Food safety	/	/	Within 20 min, the kill rate of *Enterococcus faecalis* was approximately 96%, and the kill rate of *E. coli* was approximately 98%	The scalability of the application scope is quite high	[64]
	Gold-modified graphitic carbon nitride Nanosheets 3.7 ± 1.2 nm	Sugar	Green processing	Disperse the Au/CN powder directly into the sugar water solution	Nanosheets: 0.5 mg/mL UW: 40 kHz, 160 W and ≤3 h; Continuous mode	Maximum hydrogen production rate: Glucose solution: 1533.3 μmol/g/h; Orange juice: 1568.0 μmol/g/h; Grape juice: 1385.8 μmol/g/h	The efficiency of H_2_ production is influenced by a multitude of complex factors that necessitate further research to optimize and understand the underlying mechanisms	[65]
Ozone	Polyethylene film doped with nano silver	*Agaricus bisporus*	Food preservation	Place *Agaricus bisporus* directly in a polyethylene packaging bag that contains nano-silver	Nano silver: 2.711 mg/m^3^ Ozone: 15 min	Compared with traditional commercial polyethylene packaging, the shelf life was extended by 6 to 9 days; Significantly delayed browning and softening, maintaining a high whiteness and a low browning index	The migration of nano-silver during the packaging process needs to be further evaluated	[66]
	TiO_2_ nanoparticles 35–50 nm	Cooking fumes	Green processing	Coated onto the glass fiber filter to be used for treating cooking fumes (volatile organic compounds)	/	Maximum volatile organic compounds removal rate: 94%	There may be engineering challenges in scaling up the continuous-flow reactor in the laboratory to an actual kitchen exhaust system	[67]
	Cu-doped MnO_3_ nanocatalyst	Apples and bananas	Food preservation	Catalysts loaded with nanoparticles were combined with ozone to sterilize the environment and degrade ethylene	Nanocatalyst: 6 mg/g Ozone: 40 ppb	The bactericidal efficiency against *E. coli* reached 99.9% within 24 h; Extended the shelf life by more than ten times; It effectively delayed the softening, browning and mold growth of fruits, and maintained the appearance, color and structural integrity of the fruits	The large-scale production of catalysts may encounter challenges related to cost and process stability	[68]
	Ozone micro-nano bubbles 5 nm–20 μm	Parsley	Food quality	Soak the parsley in the ozone micro-nano bubble water that has been prepared in advance; ozone concentration	Ozone: 10 min	The weight loss rate decreased by 64.1% and contents of chlorophyll and vitamin C were higher after 5 days at 20 °C, compared to the control; Its hardness was 15% higher and it also achieved a higher sensory score	In large-scale production, it is necessary to ensure the stability of the bubble generation system and the controllability of the concentration	[69]
	OMNB/SPC sterilization system 339.3 nm	Kyoho grapes	Food safety and preservation	The fruits are immersed in a water treatment tank containing OMNB and SPC particles	SPC: 10 g Ozone: 2 mg/L, 3 min	Complete inactivation of 7 log CFU/mL of *E. coli* and *S. aureus* within 20 min; The shelf life was extended to 12 days, while the appearance remained fresh	The piezoelectric properties of SPC may deteriorate over time or due to mechanical fatigue	[70]
	Ozone nanobubbles	Pork	Food preservation	Immerse the pork samples in the ozone nano-bubble solution	Ozone: 5.0 ppm, time: 1 min	For pork inoculated with pathogenic bacteria, ozone nano-bubbles can achieve a reduction of 1–2 log CFU/cm^2^	The sterilization capability is slightly insufficient	[71]
	Ozone nanobubbles	Peaches	Food preservation	Immerse the peaches in a solution of nano-bubble water containing Ozone	Ozone: 8 mg/L, time: 30 min	The high concentration of ozone caused some of the peaches to rot	Although high concentrations of ozone have strong oxidizing properties, it can cause damage to fruit tissues and is not suitable for commercial applications	[72]
Pulsed electric field (PEF)	Peppermint essential oil nano emulsion <200 nm	Guava juice and mango juice	Food safety	Add the prepared nanoemulsion directly to the juice	Nanoemulsion: 0.16–0.31 µL/mL PEF: 30 kV/cm, 1 Hz	Maximum reduction in microbial count: ≥5 log CFU/mL; Preserved the heat-sensitive nutrients and the original flavor	The addition of essential oils may still affect the flavor, and consumer acceptance testing is necessary	[73]
	Pectin nanoparticles loaded with nisin	/	Food safety	Add the pre-prepared nanoparticle suspension directly to the bacterial suspension	Nanoparticle: 0.2 mg/ML PEF: 20–30 kV/cm, 1 kHz	Under optimal synergy conditions, a reduction of approximately 2–3 log CFU/mL could be achieved	The nanosecond PEF equipment is more complex and costly for its large-scale industrial application	[74]
	Citral nanoemulsions 161–191 nm	Apple juice	Food safety	Add the prepared nanoemulsion directly to the apple juice	Nanoemulsion: 0.2 µL/mL PEF: 30 kV/cm, 150 µs	The maximum reduction in microbial count was approximately 5 log CFU/mL	The synergistic effect between PEF and citral is relatively weak	[75]
Magnetic field (MF)	Nisin-loaded magnetic nanoparticles ~11.3 nm	/	Food safety	Mix the suspension of nanoparticles loaded with nisin directly with the bacterial sample	Nanoparticle: 0.04 mg/mL MF: 125 mT, 200 kHz and 30 min	Under the optimal conditions, approximately 3 log CFU/mL of inactivation effect were achieved on *Listeria*	Complexed food components may affect the distribution of nanoparticles and the penetration of magnetic fields	[76]
	Magnetic microbead 50 μm	Liquid whole eggs	Food quality	Disperse the magnetic microbeads directly into the egg liquid	Magnetic microbead: 0.7% (*w*/*w*) MF: 400 kHz and 60 s	The *Salmonella* bacteria were inactivated by 7.6 log CFU/mL under the optimal conditions; The protein did not undergo denaturation. The apparent viscosity, foaming ability and stability were like fresh egg liquid	Micron-sized particles may be difficult to distribute evenly in food, which can result in local overheating or insufficient heating	[77]
Cold plasma (CP)	Nano-ZnO Antimicrobial Film	Grapes	Food preservation	Prepare nano-zinc oxide into antibacterial films and use them for packaging grapes	CP: 78 kV, 110 Hz and 116 s	The initial sterilization rate of the co-processing method was 99.34%; The shelf life was extended by 8 days at 4 °C; It maintained higher contents of soluble solids, vitamin C and titratable acid, and reduced the quality loss rate	For different varieties, sizes and packaging quantities of grapes, the process parameters need to be re-optimized	[78]
	Nano ZnO	Steamed fish cake	Food safety	Pack the fish cakes with packaging containing Nano ZnO	CP: 23 kV and 3 min	The maximum reduction in microbial count was 2.4 log CFU/g; The shelf life was extended to 21 days; It had no significant effect on the moisture content, water activity, color, pH, or gel strength of the sample, and the protein remained un-denatured	The synergistic effect is not obvious, and the sterilization effect is easily affected by the quantity of samples within the packaging	[79]
	Cellulose/PVA/Nanoclay (CPN) nanocomposite	Wheat flour	Food safety	Utilizing nano-composite films for packaging flour	CP: 12 kV/7 min and 15 kV/10 min	The combined treatment completely eliminated *Aspergillus flavus*; Its shelf life was extended to at least 90 days; It also effectively maintained gluten and starch contents during storage	The actual production costs may be higher than expected	[80]
	Cellulose/PVA/Nanoclay (CPN) nanocomposite	Saffron	Food preservation	The method is the same as above	CP: 6 kV/5 min and 7 kV/3 min	Complete elimination of *E. coli* could be achieved in 90 days; The shelf life was extended to at least 90 days; The key active components of saffron maintained a stable content under combined treatment, which was superior to single treatment	How the plan can be adapted to continuous and large-scale food packaging production lines remains to be verified	[81]
	Linalool nanoemulsions ~103.24 nm	Ready-to-eat chicken meat	Food safety	Immerse the chicken samples in the nanoemulsion solution	After 5 min of CP treatment, the sample was washed with nanoemulsion for 25 min	The combined treatment reduced the levels of *E. coli* O157:H7 and *Salmonella* by 2.76 and 3.24 log CFU/g respectively	The CP equipment can be further optimized to become a continuous or batch processing system	[82]
	D-limonene nanoemulsion 340 nm	Soy-based beverages	Food safety	Add the nanoemulsion directly to the beverage	Nanoemulsion: 0.05 mol/L CP: 300 W and 30 min (20% O_2_, 80% N_2_)	CP pre-treatment combined with D-limonene nanoemulsion showed a synergistic antibacterial effect	The composition of the food matrix has a crucial impact on the sterilization effect of this scheme	[83]
	Nanobubbles ~188 nm	/	Food safety	/	/	Under the optimal continuous processing, a reduction of 2.75 log CFU/cm^2^ was achieved in the mixed biofilm of *Salmonella* and *Aeromonas australiensis*	The antibacterial mechanism involves the synergistic action of multiple and complex factors, and the specific dominant mechanism is still unclear	[84]

^a^: Type of nanomaterials; Size. ^b^: Target food matrix. ^c^: Application scenario, including Food safety, Food preservation, Food quality and Green processing. They primarily encompass four objectives: (1) mitigating the harm of microorganisms to food safety, particularly by controlling pathogenic bacteria; (2) extending the shelf life of food and maintaining its sensory properties, particularly by controlling spoilage bacteria; (3) improving the processing effect of food to enhance its sensory quality; (4) reducing the adverse environmental impacts of food processing, thereby making it more environmentally sustainable and aligned with the principles of sustainable development. ^d^: The method of introducing nanomaterials. ^e^: The dosage and time of specific food processing techniques combined with nanomaterials under optimal conditions. ^f^: Application results: Quantitative data, such as the number of microorganisms, the removal rate of toxins; extension of shelf life; and improvement in sensory attributes. ^g^: Possible limitations: matrix effects, aggregation, stability or scalability (excluding the dose exposure characteristics of nanomaterials). All the information is listed only after it has been reported in references.

## Data Availability

No new data were created or analyzed in this study. Data sharing is not applicable to this article.
